# Importance of Arterial Vessel Length for Metastatic Lymph Node Retrieval and Survival in Standardized Left- and Right-Sided Colon Cancer Surgery

**DOI:** 10.1007/s12029-022-00863-7

**Published:** 2022-10-14

**Authors:** Catarina Tiselius, Csaba Kindler, Kenneth Smedh

**Affiliations:** 1 Department of Surgery, Västmanland Hospital Västerås, Västerås, Sweden; 2 Department of Pathology, Västmanland Hospital Västerås, Västerås, Sweden; 3 Centre for Clinical Research Västerås, Uppsala University, Västmanland Hospital Västerås, Västerås, Sweden

**Keywords:** Colon cancer, Lymph node metastases, Surgery, Arterial ligation, Survival

## Abstract

**Background:**

We investigated the localization of lymph node metastases, and the role of arterial vessel and specimen lengths in left- and right-sided colon cancer surgery, for survival.

**Methods:**

This was a prospective cross-sectional population-based study of specimens from patients who underwent standardized surgical resection for colon cancer in 2012–2015. The mesocolon of the specimens was divided into four sections for pathological analysis of lymph nodes. Multiple linear regression analysis was used to explore the relationship between lymph node counts and patient- and surgery-related factors. For survival analysis, a multivariable Cox regression method was used.

**Results:**

A total of 317 patients (160 females) were included. Median (range) age was 74 (30–95) years. Median number of lymph node retrieval was 32 (8–198) and was associated with increased specimen length but not to arterial vessel length. One hundred and thirty-three (42%) patients had lymph node metastases. All patients had these located < 5 cm from the tumour. Ten, two, and three specimens had lymph node metastases around the central and peripheral ligation of the ileocolic artery and at the central ligation of the inferior mesenteric artery, respectively. The tumour stages in these specimens were T3-4N2M0-1. No statistically significant survival benefit was associated with longer arterial vessel length (*p* = 0.429).

**Conclusions:**

Neither retrieval of lymph nodes nor statistically significant survival was affected by vessel length in standardized left- and right-sided colon cancer surgery.

## Introduction

During the last decades, the surgical technique for resection of colon cancer has been improved to be less traumatic, with a more central ligation of the tumour feeding vessel, and with focus on resection and preserving the intact mesocolon fascia, along with a better prognosis [1]. Moreover, the oncologic treatment has also improved with more tailored regimens in both adjuvant and palliative settings [2]. The lymph node status has a prognostic value and is of major importance in the decision to offer adjuvant treatment in patients with radically resected colon cancer. Thus, lymph node retrieval is of importance for oncological reasons. However, the optimal extent of the surgical resection from the tumour and the ligation site of the tumour feeding arterial vessel are still under debate [3].

Wide excisions and central arterial ligation of the tumour feeding vessel are recommended for greater lymph node yields and are considered quality markers for the surgical outcome [4]. However, published reviews and meta-analyses have not been able to clearly demonstrate a better oncological outcome after extended lymph node dissection [5, 6]. Furthermore, some studies have shown that there is an increased risk of postoperative complications and mortality with this type of surgery. Zeng and Su have shown in a meta-analysis that there is an increased risk for anastomotic leakage and overall morbidity in high ligation of the inferior mesenteric artery (IMA) [7]. In right-sided colon cancer, no benefit was seen with extended mesenteric resection with central vascular ligation [8] and wide excision was associated with decreased survival [9]. Furthermore, no survival benefit has been shown between high versus low arterial ligations [10, 11].

The primary aim of the present study was to investigate the anatomical location of mesenteric lymph node metastases in different parts of specimens from patients standardized operated for right- and left-sided colon cancer in relation to arterial vessel and specimen lengths. A secondary aim was to elucidate if the length of the tumour feeding vessels in right- and left-sided colon cancer surgery affected survival.

## Materials and Methods

### Patients

**Fig. 1:**
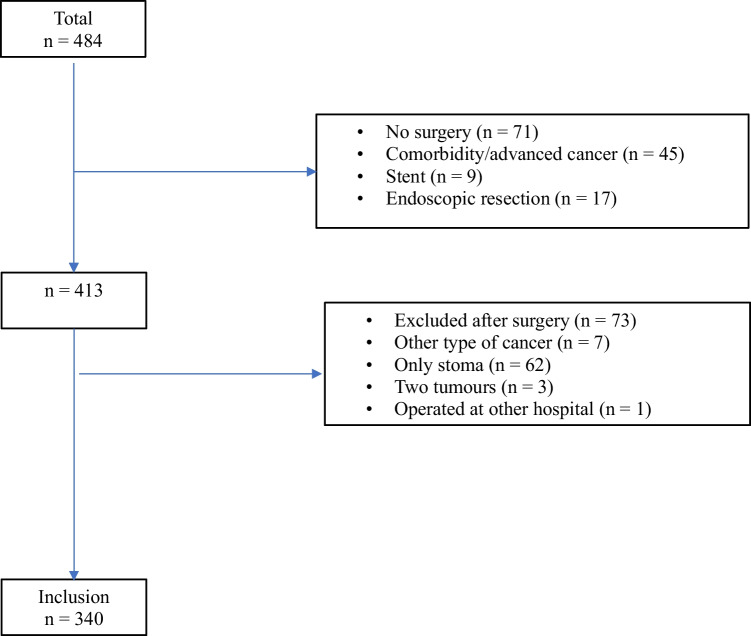
CONSORT flow diagram of the number of patients diagnosed with colon cancer in 2012–2015 in Västmanland County, Sweden

In this population-based study, all patients who underwent primary resection for colon cancer between 1 January 2012 and 31 December 2015 in Västmanland County, Sweden, were enrolled. Västmanland County Hospital is a single institution serving 270,000 people in a defined geographical area. The cohort comprised 484 patients who were registered with a colon cancer diagnosis in the above-mentioned period (Fig. 1).

The cases represented a consecutive and unselected cohort. Seventy-one patients were excluded due to severe comorbidity/advanced cancer, stenting treatment, or endoscopically removed polyp cancer. After surgery, 73 patients were excluded due to other types of cancer, stoma without tumour removal, two cancers, or surgery at another hospital. Patients with transverse colon cancer (*n* = 23) were excluded due to heterogenous resection types. Specimens from 317 patients operated for colon cancer with right or left colectomy were included (Fig. 1).

Information about patient background data such as age, sex, American Society of Anaesthesiologists (ASA) physical status classification, body mass index, tumour location, preoperative tumour stage, emergency/elective, and type of surgery were prospectively collected in case report forms and from medical records and follow-up data from the Swedish Colorectal Cancer Registry. Data were then retrospectively analysed. All patients were examined using a computed tomography scan of the chest and abdomen and colonoscopy or a computed tomography scan of the colon.

### Surgery

The emergency and elective surgery was performed by specialists at the colorectal unit. Patients underwent resection using laparoscopy or an open technique. The surgical technique was standardized with dissection along the anatomical visceral planes in order to preserve the mesocolic fascia intact. The bowel was divided with at least 5-cm margin in the proximal and distal directions of the bowel.

Right-sided tumours located at the caecum or at the ascending colon were operated on using right colectomy. Tumours located at the hepatic flexure or at the proximal part of the transverse colon were operated on using “extended” right colectomy. Left-sided tumours located at the distal part of the transverse colon and the splenic flexure or at the descending or proximal sigmoid colon were operated on using left colectomy or sigmoid resection, respectively. Distal sigmoid tumours were operated on using high anterior resection.

**Fig. 2:**
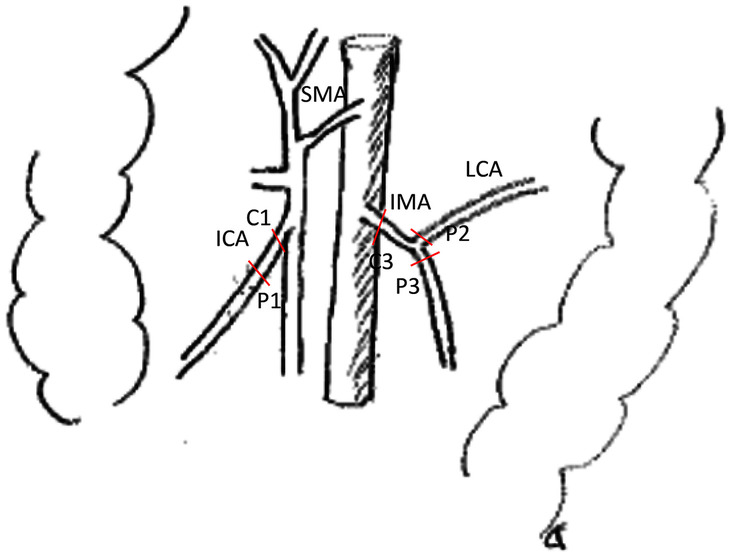
A schematic figure of central and peripheral arterial ligation sites of ileocolic artery (ICA) with central (C1) and peripheral (P1) ligation, left colic artery (LCA) with peripheral (P3) ligation and mesenteric inferior artery (IMA) with central (C4), and peripheral (P4) ligation at superior rectal artery (SRA)

Central arterial ligation was gold standard, but certain patient characteristics and tumour factors implied selection for a peripheral ligation. The ligation site was recorded in a protocol by the operating surgeon and sent to the Swedish Colorectal Cancer Registry in right- and left-sided tumours. A central ligation of the ileocolic artery (ICA) was defined as a ligation approximately within 1 cm of the superior mesenteric artery. In left-sided tumours, an arterial ligation of the inferior mesenteric artery (IMA) was either approximately 2 cm from the aorta in central/high ligation or at the superior rectal artery (SRA) close to the origin of the left colic artery in peripheral/low ligation [12]. Ligation at the base of the left colic artery (LCA) was recorded as a peripheral ligation in left colectomy (Fig. 2).

### Pathological Analysis

**Fig. 3:**
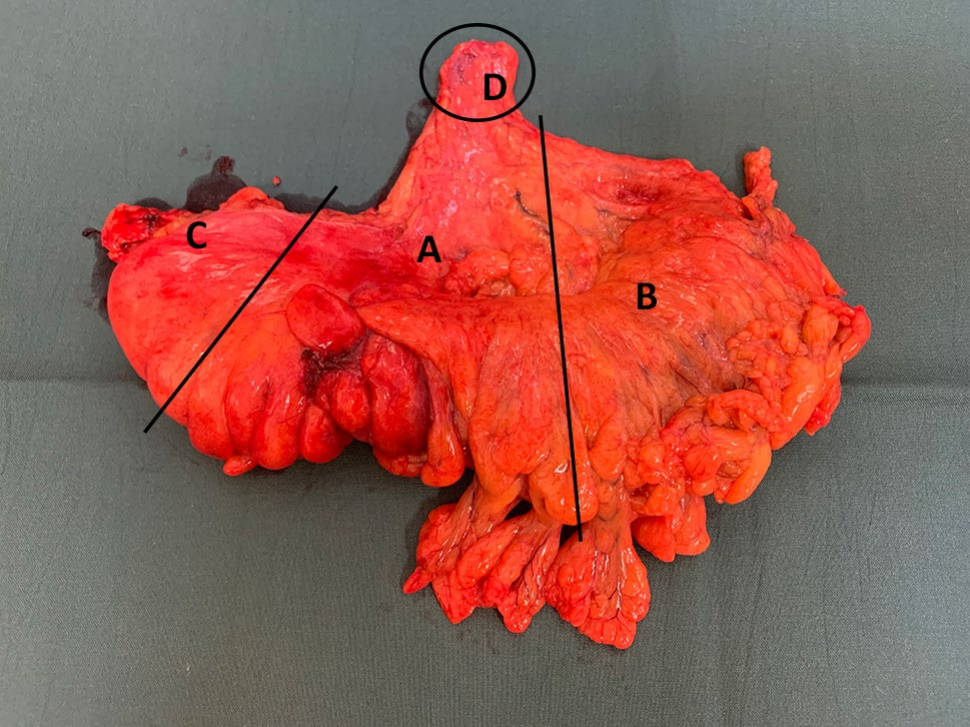
Colon specimen from a high anterior resection divided into four sections: the area around the tumour (**A**), the mesentery proximal to the tumour (**B**), the mesocolon distal to the tumour (**C**), and the area around the arterial ligation (**D**)

Resected specimens were pinned to a board and fixed in 4% buffered formalin for 72–96 h. Two experienced gastrointestinal pathologists examined and reported all gross and microscopic pathological findings. The specimens were divided into four sections according to a specific protocol (Fig. 3).

The mesocolonic fat 5 cm from the tumour and around the tumour feeding vessel (A), the mesenteric fat proximal to the colon (B), the mesocolonic fat distal to the colon (C), and the area 3 cm around the arterial ligature (D) were analysed separately for lymph node retrieval (Fig. 3). The length of the tumour-feeding artery was measured from the ligation site to the bowel wall close to the tumour.

The lymph nodes were dissected via manual palpation. A Fatclearance technique was used. All lymph nodes were stained using haematoxylin and eosin and were examined for the presence of tumour metastasis using light microscopy. Each specimen was cut in parallel sections, approximately 5 mm thin. Each dissected lymph node was cut in 2 sections for analysis. Tumours were assessed for histological type, pT stage, pN stage, tumour differentiation, lymphatic vessel invasion, venous invasion, and perineural invasion.

Histologic diagnoses were based on the *TNM Classification of Malignant Tumours*, 7th Edition [13]. The pathologists graded the tumour as a T4 tumour if the tumour grew into the serosa.

### Survival

Overall and disease-free survival was calculated using the Kaplan–Meier method and the Cox regression method for patients with radically operated colon cancer and tumour stages I–III and with ASA status 1–3. Overall survival time was defined as the time from surgery to death from any cause and disease-free survival as the time from surgery to local or distant recurrence. Data were selectively analysed for right- or left-sided colon cancers according to the tumour arterial feeding vessel being the ICA or LCA and IMA. The possible factors affecting survival such as age, sex, body mass index, comorbidity (ASA class 1–3), tumour stage (TNM I–III), and vessel length were included in the multivariable regression analysis.

### Ethics

The study was approved by the Ethics Committee of Uppsala University (Dnr 2013/099) and registered at ClinicalTrials.gov (NCT03314961).

### Statistical Analyses

Categorical data are presented as frequencies and percentages, *n* (%), while continuous data are presented as means and standard deviations (SDs), supplemented with median (range). Tests of differences between categorical variables were performed using Pearson’s *χ*^2^ test or Fisher’s exact test. Multiple linear regression analyses were used to examine how the number of lymph nodes and documented central versus peripheral arterial ligation were associated with patients’ age, sex, body mass index, emergency/elective surgery, vessel length, and specimen length. Relative risk was calculated using 95% confidence intervals (CIs). The possible determinants were tested in a multivariable Cox proportional hazards regression model, wherein the hazard rate ratio was considered an estimate of the relative risk. Kaplan-Meyer analysis was performed to assess overall and disease-free survival in right and left colectomies. For all analyses, a *p*-value < 0.05 was considered statistically significant. All statistical analyses were performed using IBM SPSS Statistics, version 26 or later (IBM SPSS, Armonk, NY, USA).

## Results

The detailed demographic and clinical characteristics of patients operated for right- and left-sided colon cancer in 2012–2015 are listed in Table 1. Patients with right-sided tumours were older, were more often females, had higher comorbidity (ASA status), more often had a documented central ligation of ICA, and had a less advanced tumour stage.

**Table 1:** Demographic and clinical characteristics of the patients operated for colon cancer in 2012–2015 (*n* = 317)

Variable	Right-sided *n* = 188 Value	Left-sided *n* = 129 Value	*P*-value
Age (years), mean (SD)	75.2 (10.4)	69.2 (11.5)	< 0.001
Sex, *n* (%)			0.023
Male	83 (44)	74 (57)	
Female	105 (56)	55 (43)	
BMI (kg/m^2^), mean (SD)	26.5 (4.0)	27.0 (5.3)	0.287
ASA status, *n* (%)			0.026
1	14 (7.4)	22 (17.1)	
2	102 (54.3)	65 (50.4)	
3	65 (34.6)	41 (31.8)	
4	7 (3.7)	1 (0.8)	
Laparoscopy/open surgery, *n* (%)			0.540
Laparoscopy	29 (15.4)	24 (18.6)	
Open surgery	159 (84.6)	105 (81.4)	
Emergency/elective surgery, *n* (%)			0.290
Emergency surgery	31 (16.5)	31 (24.0)	
Elective surgery	157 (83.5)	98 (76.0)	
Ligation type* (all surgeries)			0.006
Central	175 (93.1)	107 (82.9)	
Peripheral	13 (16.9)	22 (17.1)	
Pathological T stage, *n* (%)			0.004
1	4 (2.1)	13 (10.1)	
2	33 (17.6)	13 (10.1)	
3	55 (29.3)	30 (23.3)	
4	96 (51.1)	73 (56.6)	
Pathological N stage, *n* (%)			0.312
0	115 (61.2)	66 (51.2)	
1	39 (20.7)	41 (31.8)	
2	34 (18.1)	22 (17.1)	
Pathological M stage, *n* (%)			0.616
0	164 (87.2)	110 (83.5)	
1	24 (12.8)	19 (14.7)	
Pathologic stage, *n* (%)			0.001
1	4 (2.1)	11 (8.5)	
2	102 (54.3)	43 (33.3)	
3	60 (31.9)	57 (44.2)	
4	22 (11.7)	18 (14.0)	

A total of 84% (264/317) of the surgeries were performed using an open technique. One-third (111/317) of the patients who underwent surgery with resection received a permanent or diverting stoma for safety reasons due to emergency surgeries (21/111; 19%), high comorbidity/ASA status 3–4 (41/111; 37%), or poor sphincter function. Multiple linear regression analyses were used to examine how the surgeons’ documented central versus peripheral arterial ligation was associated with patients’ age, sex, body mass index, ASA class, emergency/elective surgery, and TNM stage. The analysis showed that patients with more comorbidity and a more advanced TNM stage more often had a documented peripheral arterial ligation *β* (95%CI); −0.07 (−0.13 − (−0.01)), *p* = 0.027, and −0.08 (−0.12 − (−0.02)), *p* = 0.003, respectively.

Tumour location and the types of surgeries as well as data on median (range) of vessel-, specimen length, the median proximal, and distal bowel resection length are presented in Table 2. Data are sorted by the tumour feeding vessels; ileocolic artery (ICA) with central (C1) and peripheral (P1) ligation, left colic artery (LCA) with peripheral (P2) ligation, and mesenteric inferior artery (IMA) with central (C3) and peripheral (P3) ligation at the superior rectal artery (SRA). Most patients were operated using either right colectomy (188/317, 59%) or high anterior resection (105/317, 36%).

**Table 2:** Demographic and clinical characteristics of the 317 patients operated for colon cancer sorted by the four tumour feeding vessels and central and peripheral ligation sites, respectively: ileocolic artery (ICA) with central (C1) and peripheral (P1) ligation, left colic artery (LCA) with peripheral (P3) ligation, and inferior mesenteric artery (IMA) with central (C4) and peripheral (P4) ligation of superior rectal artery (SRA). Descriptive data on arterial vessel length and specimen length are also presented

**Colon cancer**	**Right sided**		**Left sided**		
**Vessel type**	**Ileocolic artery (ICA)**		**Left colic artery (LCA)**	**Inferior mesenteric artery (IMA)**	**Superior rectal artery (SRA)**
**Ligation type**	**Central (C1)**	**Peripheral (P1)**	**Peripheral (P2)**	**Central (C3)**	**Peripheral (P3)**
**Number**	175	13	24	85	20
**Tumour location**					
Caecum	93	8			
Ascendence	54	5			
Hep. flexure	17		1		
Prox. transvers	11				
Dist. transverse			4	1	
Lien flexure			11	1	
Descendence			6	10	3
Sigmoid			2	73	17
**Surgery type**					
Ileocaecal resect	1	2			
Right	165	11		1	
Ext. right	7		1		
Left			21	12	
Colectomy	2		1	8	2
Anterior res			1	65	18
**Vessel length** **Mean (SD) cm**	8.5 (1.98)	6.8 (2.21)	7.4 (2.0)	10.1 (2.69)	8.0 (1.71)
**Median (range) cm**	9 (3–13)	7 (3–13)	8.0 (4–10)	10.0 (3–16)	8 (5.5–11)
**Number**	165	11	15	73	15
**Specimen length cm**	31.8 (11.5)	27.0 (7.20)	19.0 (1.41)	32.3 (20.1)	29.8 (18.0)
**PBL** **Mean (SD) cm**	10.7 (7.6)	9.0 (5.9)	9.5 (6.4)	16.3 (18.0)	14.5 (9.9)
**DBL** **Mean (SD) cm**	15.7 (8.2)	13.1 (5.7)	7.0 (5.7)	11.9 (7.3)	10.0 (8.4)

*DBL* distal bowel resection length, *LN* lymph node, *PBL* proximal bowel resection length, *SD* standard deviation

*n* (SD) = mean number (SD)

All patients underwent local radical resection (R0) with CRM mean (SD) of 20.6 (22.6) mm and median of 10 (1–150) mm.

In Table 3 data are sorted in columns as in Table 2. Data on total amount of lymph nodes (I), number of patients with lymph node metastases (*n* = 133) (II), total number of lymph node metastases (*n* = 628), (III) divided by part of specimen (A–D). The total median (range) number of retrieved lymph nodes was 32 (8–198).

**Table 3:** Descriptive data on lymph nodes (LN) (I–III) sorted by the three tumour feeding vessels and central and peripheral ligation sites, respectively: ileocolic artery (ICA) with central (C1) and peripheral (P1) ligation, left colic artery (LCA) with peripheral (P2) ligation, and mesenteric inferior artery (IMA) with central (C3) and peripheral (P3) ligation at superior rectal artery (SRA)

**Colon cancer**	**Right sided**		**Left sided**		
**Vessel type**	**Ileocolic artery (ICA)**		**Left colic artery (LCA)**	**Inferior mesenteric artery (IMA)**	**Superior rectal artery (SRA)**
**Ligation type**	**Central (C1)**	**Peripheral (P1)**	**Peripheral (P2)**	**Central (C3)**	**Peripheral(P)3**
***N* patients**	175	13	24	85	20
**Mean (SD) LN**	33.5 (13.5)	31.9 (13.2)	15.5 (2.1)	37.6 (24.2)	30.6 (12.1)
***n* patients**^**~**^	170	11	23	84	20
(I) Mean number of lymph nodes in specimen parts A–D					
**Mean *n* LN**^**(I)**^**:**					
**A mean *n* (SD)**	18.5 (8.6)	22.1 (6.4)	11.0 (2.8)	21.0 (9.4)	20.0 (12.6)
**B mean**	4.8	0.8	1	6.9	5.6
**C mean**	7.3	9.7	2.5	7.2	4.25
**D mean**	3.3	2.4	1	2.2	1.9
(II) Number of patients (%) with lymph node metastases in A–D					
***n* (%) of patients with LN metastases**^**(II)**^	61/170(36)	8/11(73)	14/23(64)	42/84(49)	9/20(50)
**A**	59(100)	8(100)	15(100)	42(100)	9(100)
**B**	5(8)	1(12)	3	2(5)	0
**C**	14(23)	2(25)	1	2(5)	1(10)
**D**	10(16)	2(25)	0	3(7)	0
(III) Total number of lymph node metastases in A–D					
**Sum LN met **^**(III)**^	322	56	29	158	63
**(*n* = 628)**					
***n* (%)**					
**LN met A**					62 (98)
**(*n* = 517)**	253 (79)	39 (70)	29 (100)	134 (85)	
***n* LN met B**	9 (2.8)	1	0	2(1.2)	0
***n* LN met C**	36 (11.2)	9	0	19(11.3)	1
***n* LN met D**	24 (7.5)	7	0	3(1.8)	0

*n* patients^~^  = Number of patients with division of lymph nodes in specimen

*n* (SD) = mean number (standard deviation)

A, specimen including tumour; B, specimen proximally to the tumour; C, specimen distally to the tumour

(I) = mean total number of lymph nodes at each part A-D of the specimen

(II) = number of patients with lymph node metastases

(III) = number of lymph node metastases; LN met A = total number lymph node metastases at A–D

In all the specimens with lymph node metastases, these were present in the central specimen section around the tumour (A), and in 11% (15/133) of all cases, there were lymph node metastases around the arterial ligation site (D). Analysis of these cases showed that 14/15 were T4, 15/15 were N2, and 10/15 were metastasized (M1). In only three patients 2.2% (3/136), lymph node metastases were located solely more proximally (B) and distally (C) than in the close 5-cm section (A). In two of the three cases, lymph node metastases were only found proximal to the tumour (B), and in the third case, micro-metastasis was found in the distal section (C). Significantly more lymph node metastases were located distally to the tumour (C) in right-sided cancers; *p* = 0.039.

Data on summarized numbers of lymph node metastases (Sum LNmet.) (*n* = 628) sorted by vessel type and central versus peripheral ligation are also shown in Table 3. The data indicate that 82% (517/628) of the total number of lymph node metastases were located in the mesocolon around the tumour (A) and 2.4% (15/628), 10.5% (66/628), and 5.4% (34/628) were located proximally (B), distally (C), and around the arterial ligation site (D), respectively.

Sixty-one (19.2%) of the specimens had tumour deposits. Lymph vessel, venous, and nerve invasions were detected in 141 (44.5%), 114 (36.0%), and 49 (15.5%) specimens, respectively. Forty specimens were microsatellite instability positive, of which 31 specimens were from patients who were operated on using right hemicolectomy.

Multiple regression analysis (Table 4) showed that the total number of retrieved lymph nodes was greater with longer specimen lengths, both at arterial ligation with the ileocolic artery (ICA) and inferior mesenteric artery (IMA) in right- and left-sided colon cancer. The total number of lymph nodes was also greater in younger patients with ligation of the ICA and in male patients in high anterior resection with ligation of the IMA. The vessel and specimen lengths were longer in elective surgeries with ligation of the ICA and IMA, respectively.

**Table 4:** Multiple linear regression analyses of the relationships between (i) age, sex, BMI, and acute or elective surgery and (ii) total number of lymph nodes, vessel (ileocolic artery (ICA) and inferior mesenteric artery (IMA)) length and specimen length

	Ileocolic artery		Inferior mesenteric artery	
	*β*	*p*-value	*β*	*p*-value
	Total number of lymph nodes *n* = 175		Total number of lymph nodes *n* = 84	
Age	–0.273	0.006	–0.13	0.487
Sex	–0.027	0.989	–9.53	0.035
BMI	0.086	0.737	0.614	0.107
Emergency/elective surgery	2.46	0.373	3.48	0.562
Specimen length	0.334	< 0.005	0.971	< 0.005
Vessel length	0.158	0.849	0.244	0.760

	Vessel length *n* = 176		Vessel length *n* = 88	
Age	0.022	0.144	–0.009	0.741
Sex	0.204	0.519	–0.840	0.181
BMI	0.077	0.052	0.060	0.262
Emergency/elective surgery	1.175	0.006	–0.307	0.718

	Specimen length *n* = 186		Specimen length *n* = 101	
Age	–0.14	0.094	–0.164	0.325
Sex	–3.53	0.036	0.407	0.917
BMI	0.040	0.850	–0.236	0.492
Emergency/elective surgery	0.280	0.902	–17.85	< 0.005

*β* regression coefficient, *BMI* body mass index

*p*-value < 0.05 considered significant

### Follow-up/Survival

**Fig. 4:**
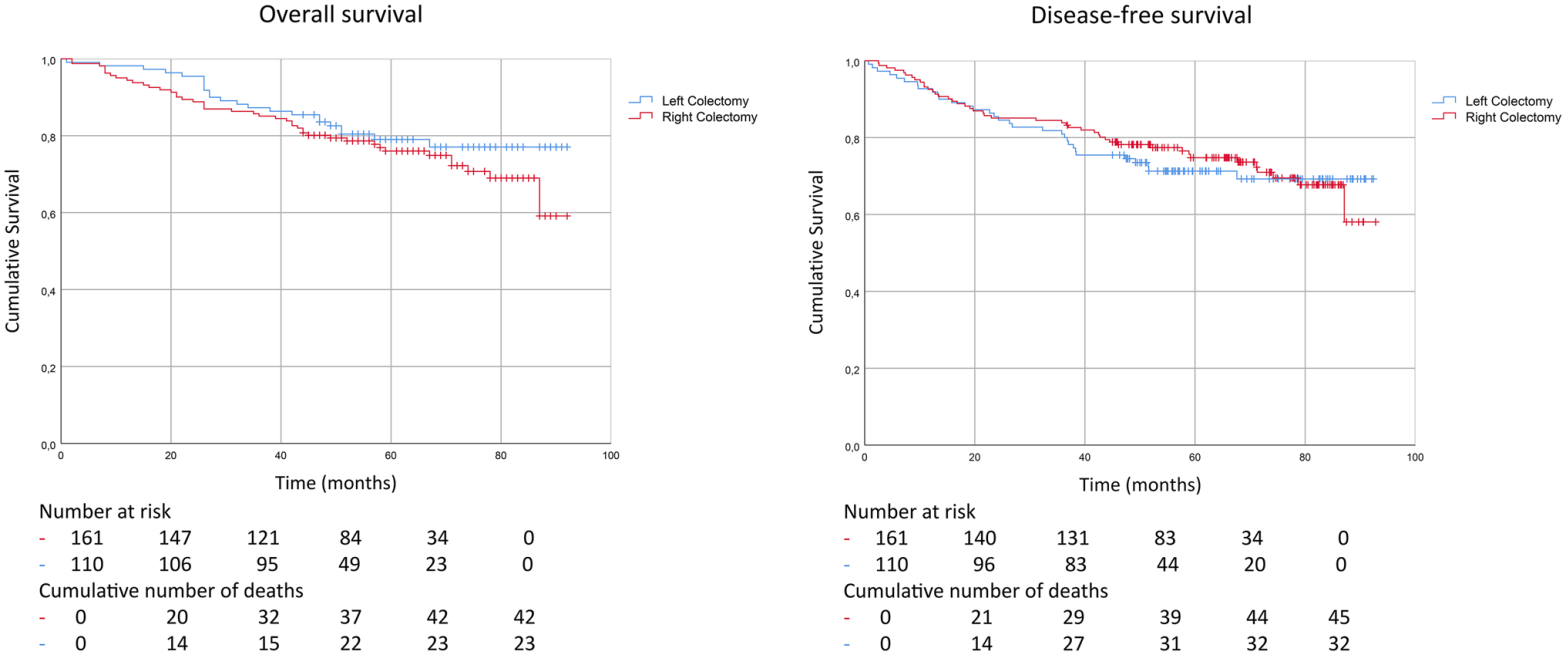
Overall and disease-free survival. Kaplan–Meier analysis of overall survival A and disease-free survival B of patients with non-metastatic colon cancer (TNM I-III) and with less comorbidity (ASA status 1–3) of colon cancer patients, with right or left colectomy

The mean (SD) follow-up time was 54.7 (24.7) months and median (range) follow-up time was 57 (0–92) months. The Kaplan–Meier distributions for overall survival (OS) and disease-free survival (DFS) for right- and left-sided colon cancer are shown in Fig. 4. The 3-year overall survival was 87.3% and 85.1% (*p* = 0.609) and DFS was 80.9% and 83.9% (*p* = 0.522) in right- and left-sided colon cancers, respectively. The multivariable Cox proportional hazards regression analysis did not show a statistically significant survival benefit for longer tumour feeding arterial vessels (Table 5).

**Table 5:** Cox regression analysis of overall survival and disease-free survival of patients with, non-metastatic colon cancer (TNM I-III) and with less comorbidity (ASA status 1–3), right- or left-sided colon cancer

	Overall survival		Disease-free survival	
	HR (95% CI)	*p*-value	HR (95% CI)	*p*-value
Age	1.08 (1.04; 1.12)	< 0.001	1.05 (1.016; 1.081)	0.003
Gender	0.86 (0.50; 1.49)	0.596	0.98 (0.593; 1.620)	0.938
BMI	0.99 (0.934; 1.05)	0.769	1.02 (0.963; 1.069)	0.582
ASA (1–3)	1.31 (0.786; 2.176)	0.302	1.14 (0.719; 1.808)	0.578
TNM stage (I–III)	1.84 (1.132; 3.00)	0.014	2.02 (1.286; 3.159)	0.002
Vessel length	0.97 (0.868; 1.092)	0.643	0.95 (0.856; 1.062)	0.389

*HR* hazard ratio, *BMI* body mass index, *ASA* American Society Anaesthesiology Association, *TNM* tumour node metastasis

## Discussion

This study shows that in all of the patients with standardized resected colon cancer with lymph node metastases, the metastases were in the mesocolon 5 cm lengthwise around the tumour and along the arterial feeding vessel. Lymph node metastases were located at the ligation site in 15 cases (11%). These patients had more advanced tumours. Lymph node metastases were located more distally along the bowel in 3 cases, but this affected the accuracy of pathological tumour staging in less than 2% of cases. The results showed that the total number of lymph nodes was affected by specimen length, but not the amount of lymph node metastases. No overall survival benefit was associated with longer tumour feeding arterial vessel length in standardized resected right- or left-sided colon cancer. This study provides data in the context of the time-consuming analysis of lymph nodes in the specimen.

For proper management of patients with colorectal cancer, accurate staging is critical, and subsequently, retrieval and analysis of lymph nodes. The number of lymph nodes obtained in resected colorectal cancer specimens is dependent on clinicopathological factors such as tumour location, surgical technique [14–16], as well as quality of the pathology [17]. There is a gold standard in colorectal cancer surgery that at least 12 lymph nodes should be analysed in colorectal cancer specimens [18, 19]. This standard has been challenged by recent studies. Del Paggio et al. have showed a significant increase in the risk of death from cancer in cases with a yield below 20 lymph nodes for stage II disease [20]. In our study, using standardized surgical technique, the mean number of lymph nodes was 32, far above the critical numbers. Bertelsen et al. [21] have in a recent cohort study compared surgical technique with complete mesocolic excision (CME) with conventional resections. The group with CME had a lymph node yield of 38 as compared to 21 in the conventional group. They reported a reduced risk of recurrence and improved long-term outcome after surgery with CME.

The prognostic importance of the width of segmental resection has been studied in a randomized clinical trial of colon cancer. That study could not demonstrate any difference in survival or early morbidity in left colectomy versus segmental resection [22], and this finding is supported by a more recent study [23]. In contrast, Cserni et al. report that only 3 cm of bowel length proximal and distal from the tumour is needed in colorectal cancer surgery to retrieve over 99% of the lymph node metastases in the specimen [24]. Pusztaszeri et al. [25] used a cut edge of 5 cm in both directions to analyse 345 colorectal cancer specimens. They found that only one colon cancer patient, devoid of lymph node metastasis in the close section, had lymph node metastasis in the distant section.

In our study, the mean proximal and distal specimen lengths were over 10 cm in each direction and Cox regression analysis showed no survival benefit in patients with longer specimens (data not shown). Overall, in 98% of the cases, the lymph node metastases were in the 5-cm close section. In only three patients, lymph node metastases were solely located more proximally and distally than in the 5-cm close section. Nevertheless, we could conclude that the accuracy of pathological staging was 98% if only the analysis of the close section together with the area for the tumour feeding vessel was performed. This needs to be considered in decision of the extent of necessary resection margins, since extensive surgery is associated with more significant complications, especially in the growing portion of old, comorbid, and frail patients.

This study showed lymph node metastases around the ligation site of the arterial tumour feeding vessel in 11% of cases. However, these cases had more advanced tumour stages and the finding did not alter pathological staging. Our previous study on rectal specimens, using the same analysis protocol, was not able to demonstrate this [10]. However, most of these patients received radiotherapy and the anatomy differed with regard to the rectum location, partly being extra-abdominal, in the pelvis. The lymphatic drainage is more condensed in the rectum than in the colon with its widespread mesentery.

The role of central versus peripheral arterial vessel ligation has drawn much attention in the management of colorectal cancer surgery [5, 26–28]. However, few studies have reported the number and status of retrieved lymph nodes around the area of ligation of the ICA or IMA in right and left colon cancer surgery. The definition of the area of tissue that should be included varies [29] and there is an anatomical vessel variation [30, 31] leading to heterogeneous data. However, the mean difference in vessel length between central and peripheral ligation was very small in this study. In left colectomies this was due to that the arterial vessels were ligated close to the origin of the LCA in both central and peripheral ligation, but on different sides.

The strengths of this study are that it was population based with prospectively collected data, and surgery was standardized by colorectal surgeons with two experienced pathologists performing all the pathological analysis, without knowing if the surgeon determined the ligation site as central or peripheral. In addition, compared with other studies, a greater number of lymph nodes was retrieved by the pathologist and the specimen length was sufficient as noted above. This is also the first study that compared pathologic outcomes regarding amount and location of lymph node metastases in different sections with regard to site of arterial ligation, arterial vessel, and bowel lengths in specimens from right- and left-sided colon cancer surgery.

One weakness of this study is that it included data from only one hospital with a limited number of patients. Most surgeons determined that a central ligation was performed, and this was also the hospital policy, but this can be a subjective assessment. According to our policy and study data, there was a selection bias because patients with high comorbidity and more advanced tumour stages more often had a peripheral ligation and therefore the sample size was not powered to compare the two groups. However, this might also be the case in other non-randomized studies. Instead the important vessel length was used and the difference in cm between central and peripheral ligation was very small. The study population was somewhat heterogeneous with emergency cases included, but these constituted 20% of patients and were operated daytime by colorectal surgeons. Ideally, a postoperative angiography should have been performed to measure the remaining vessel lengths.

In this study, the overall survival of patients with longer arterial vessels, as in central ligation, was analysed after excluding patients with high comorbidity (ASA status 4) and tumour stage IV and no statistically significant survival benefit was seen.

## Conclusion and Recommendations

This study showed that there was a small variation in mean vessel length, although 88% of vessels were documented as centrally ligated in the registry. Lymph node metastases were located at the ligation site only in cases with more advanced tumour stages. Lymph node metastases were located more distally along the bowel in a few cases, but this affected the accuracy of pathological tumour staging in less than 2% of cases. There was no statistically significant survival benefit for patients with specimens with slightly longer arterial vessels as in centrally ligated vessels in this study for patients with standardized resection surgery for right- and left-sided colon cancer. Randomized trials are needed to further confirm these findings.

## Data Availability

The datasets generated during and/or analysed during the current study are available from the corresponding author on reasonable request.
